# Temporal and spatial distribution of trace metals in the Rufiji delta mangrove, Tanzania

**DOI:** 10.1007/s10661-018-6707-2

**Published:** 2018-05-09

**Authors:** Andrew Minu, Joyanto Routh, Mårten Dario, Mario Bilosnic, Rikard Kalén, J. Val Klump, John F. Machiwa

**Affiliations:** 1 Department of Aquatic Sciences and Fisheries, University of Dar es Salaam, Box 35064, Dar es Salaam, Tanzania; 2 Department of Thematic Studies-Environmental Change, Linköping University, SE-58183 Linköping, Sweden; 3 School of Freshwater Sciences, Great Lakes WATER Institute, 600 E. Greenfield Ave., Milwaukee, WI 53204 USA

**Keywords:** Trace metals, Sequential extraction, Sediments, Geoaccumulation, Enrichment factor

## Abstract

Spatial and temporal distribution of trace metals and their cycling is a key issue for understanding the ongoing biogeochemical processes in coastal environments. Sediment cores were collected from six different sampling locations from the Rufiji delta mangrove forests in southeastern coastal Tanzania that are perceived to be impacted by urban development and agricultural activities in the catchment, and pollution in upstream sections of the Rufiji River. The chronology and sediment accumulation rates at these sampling sites were derived based on the distribution of ^210^Pb_excess_ method. The trace metals (As, Cd, Cr, Cu, Ni, Pb, and Zn) were sequentially extracted as per the BCR method and analyzed. The results indicate that the mass accumulation rates range from 0.40 g cm^−2^ year^−1^ (cores NR3 and NR4) to 1.75 g cm^−2^ year^−1^ (core SR1). Trace metals in the cores are mainly associated with the residual phase and their abundances in sediments are ranked as Cr > Zn > Ni > Cu > Pb > Cd. The results imply that trace metals in the Rufiji delta mangroves are mainly of crustal origin, and they are less sensitive to weathering. Further, these metals are least available for uptake by plants and they pose limited threat to the biota.

## Introduction

Mangrove forests located in the inter-tidal zones of tropical and sub-tropical regions are both ecologically as well as economically important coastal ecosystems. The indirect and the most important value of mangroves lie in their protective role (Marchand et al. 2012; Ranjan et al. 2013). Mangrove forests help in protecting the coastlines by preventing erosion and loss of land cover by depositing large quantities of silt. The trees with their mat-like pneumatophore roots have high binding capacity for fine-grained clay and silt-rich sediments. Notably, mangrove sediments are characterized by their high organic matter (OM) content, poor nutrient quality, high amount of sulfide, and low oxygen levels (Ranjan et al. 2013). This makes mangrove sediments a sink (Aderinola et al. 2009), or for that matter even a source for various metal-bound particles that actively participate in the ongoing biogeochemical reactions (Marchand et al. 2012). Metal concentrations in these sediments depend on sediment particle size and stability of sulfide-rich minerals under anoxic conditions. Moreover, depending on the pore water conditions (Eh-pH, temperature, and salinity), these metals can transform their oxidation state, and concentrate in plant tissues resulting in long-term damaging effects (Marchand et al. 2012). Mangroves acting as a sink for metals may thus pose a threat in future through the release of these metals into the environment on decomposition of mangrove vegetation and/ change in physicochemical conditions. Hence, there is a chance of these mangroves could become a secondary source of various metal pollutants. However, trace metals mostly accumulate in surface sediments temporarily, and they are remobilized and/or re-suspended into the water column under varying environmental conditions. Destruction of mangrove forests can therefore promote remobilization of metals trapped in sediments and facilitate their transport to adjacent areas. These studies on biogeochemical processes have resulted in a keen interest about metal cycling in mangrove ecosystems (Mremi and Machiwa 2003; Marchand et al. 2012; Rumisha et al. 2012; Mrutu et al. 2013; Ranjan et al. 2013).

Tanzania accounts for ca. 0.90% of the mangrove vegetation cover worldwide. They are spread over an area of about 1155 km^2^ in a N-S transect along the Indian Ocean coast. In the past, there have been some investigations on mangroves in the Rufiji delta in Tanzania. For example, Paul and Oliver (2005) reported the case of substantial mortality in mangrove trees in the delta following severe and prolonged El-Nino events. Tafe (1990) compared the distribution of zooplankton in terms of different salinity gradients in mangrove stands. However, very little is known about distribution of trace metals or its cycling in the Rufiji delta. Largely, natural processes (weathering and erosion) and human activities (urbanization, discharge from upstream sections, and agriculture) in the catchment drive these changes. Hence, the primary objectives of the present study are to assess the distribution and mobility of trace metals in the Rufiji delta mangroves using the BCR sequential extraction protocol of the Standards, Measurement and Testing Program (formerly the Community Bureau of Reference of the European Commission) to trace the accumulation of metals sorbed to different sediment fractions. Secondly, we want to establish if accumulation of trace metals (e.g., Cd, Cr, Cu, Pb, and Ni) in the Rufiji sediments poses a threat to the flora due to their accumulation in the readily exchangeable sediment fraction. Hence, our focus is on the upper 200 cm in these cores retrieved from mangrove sites (dating back to the early 1800s) to trace the anthropogenic changes in the Rufiji delta, and its effects on the environment. To the best of our knowledge, the study provides the first broad overview about trace metal distribution in the Rufiji delta, which will be helpful for management practices to control pollution and/or launching suitable remediation practices.

## Materials and methods

### Study area

**Fig. 1:**
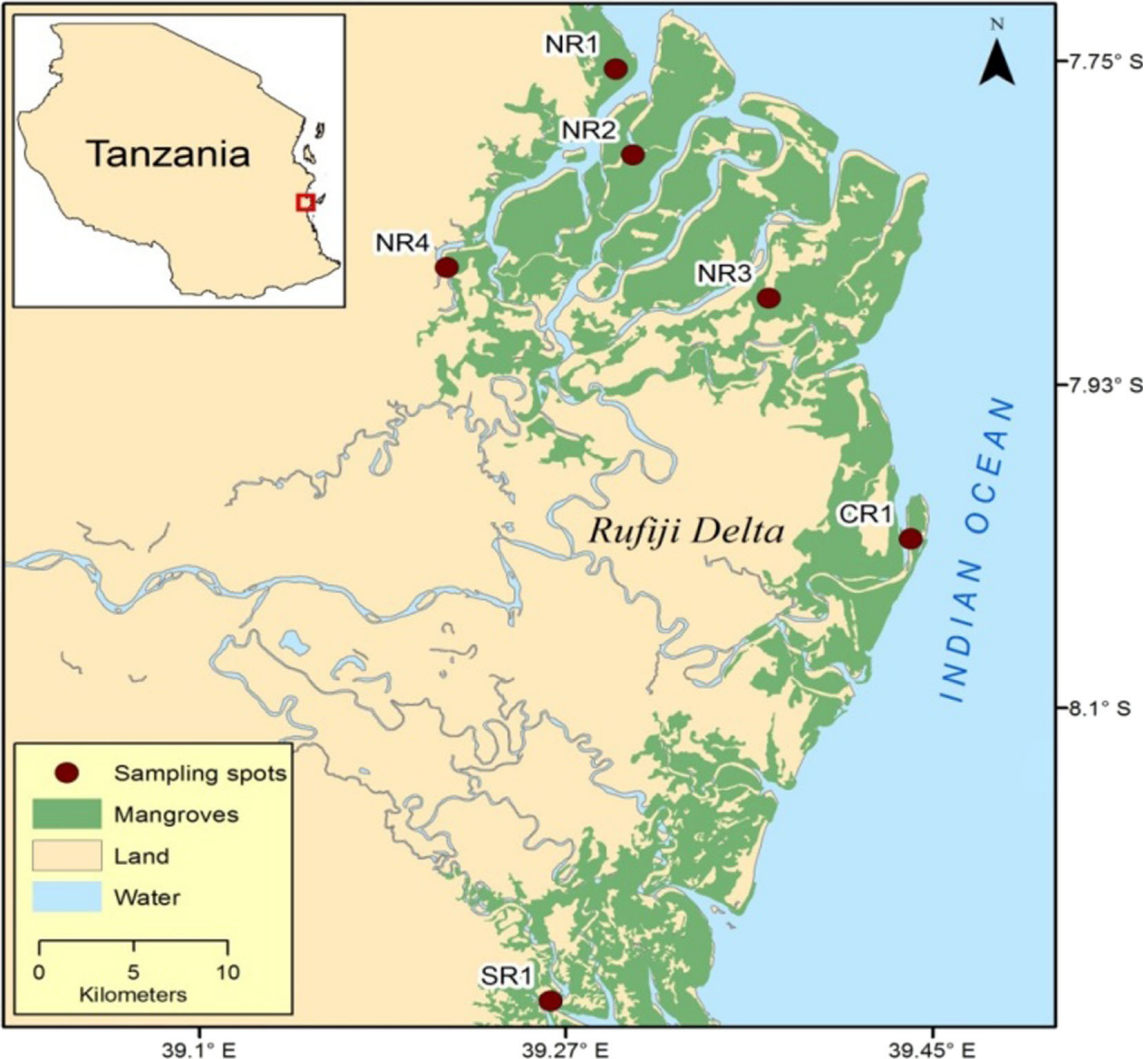
The Rufiji mangrove delta in Tanzania showing the sampling stations (modified from Google map)

The study area is in the Rufiji delta, 100 km south of Dar es Salaam. The Rufiji River has a catchment spread over 177,400 km^2^ and is over 640 km long (UNEP 2001). Nearly 30 km from the coast, the lower stretches of the Rufiji River forms a series of channels leading into a delta. A large section of the delta is covered by mangrove forests extending from 39°07′E, 7°41′S to 39°45′E, 8°15′S, which in fact forms the largest stretch of mangrove forests on the east African coast (Kilimwiko 1997). It is linked to the interior river system by an extensive flood plain. The mangrove stand covers an area of 480 km^2^, which is about 42% of the total area covered by mangrove forests in Tanzania (Fig. 1). Four tributaries discharge freshwater into the northern part of the delta. The southern part of the delta receives less freshwater and is more saline and affected by tides. Eight mangrove species occur in the Rufiji delta forests, of which, *Rhizophora mucronata*, *Avicennia marina*, and *Heritiera littoralis* are the dominant species. Other species include *Bruguiera gymnorrhiza*, *Ceriops tagal*, *Lumnitzera racemosa*, *Sonneratia alba*, and *Xylocarpus granatum* (Semesi 2002)*.* Soil type in the Rufiji delta is mostly black loamy soil and rice is cultivated widely. Large sections in upstream areas and within the delta have been cleared for rice cultivation (Taylor et al. 2003). Pesticides and fertilizers are widely used to increase agriculture output (Taylor et al. 2003). In addition, the upstream section in Rufiji River has many small towns and local industries with poor sanitation facilities that possibly contribute to the pollution in this relatively pristine area. Lax environmental regulations and poor enforcement of existing laws (Byers et al. 2012) further complicate the situation.

### Sampling

We selected six sampling stations in mangrove forests based on the dominant vegetation cover, tidal hydrodynamics, and closeness to areas with potential anthropogenic waste discharge outlets (Fig. 1). The sampling site in the northern part of the delta included station NR1 (*R. mucronata* dominated), station NR2 (*A. marina* and *A. alba* dominated), and station NR3 (*A. marina* dominated). The NR3 site differed from the other sampling stations because it is a semi-desert. The area consists of dry flat land with small prop roots sticking out in air from the soil zone. The core retrieved from NR3 was black and had strong odor of hydrogen sulfide. Station NR4 (*H. littoralis* dominated) had very hard sticky clay and fine-sand. In central part of the mangrove forest, we had one sampling station (CR1) that had *R. mucronata* as the dominant vegetation cover. Large prop roots occur all around this area and *Sonneratia* trees have been selectively logged in the forest because of its high value. The southern sampling station (SR1) is dominated by *S. alba* and *A. marina* species. This area gets inundated during high tides and remains partly submerged.

A Russian peat corer was used to retrieve the sediment cores by pushing the corer through the sediment column and transferring them to longitudinally sliced PVC pipes (50 cm long and 5 cm diameter). The core slices were wrapped in aluminum foil and plastic sheet and transported to the laboratory in cooler boxes. Intact sediment cores were obtained at 50 cm intervals, and the total core lengths at these six sites ranged from 200 to 650 cm in length. The sediment cores were sliced at 1 cm interval for the first 20 cm in each core and thereafter, the rest of the core was sliced at 2 cm interval with a stainless-steel saw. The outside edges of each slice are removed using a titanium knife to avoid any possible contamination (Givelet et al. 2004). The sediments were freeze-dried and used for various geochemical analyses that are described below.

### Reagents

All reagents were of analytical reagent grade unless otherwise stated. Double deionized water (Milli-Q water) was used for all dilutions. All standards, reagent solutions, and samples were kept in polyethylene containers. Acetic acid (glacial, 100% Fisher Scientific, UK), hydroxylammonium chloride (99% ACROS Organics, USA), hydrogen peroxide (30% Fisher Scientific, UK), and ammonium acetate (99.99%) and HNO_3_ (65%) (from Suprapur Merck, Germany) were of super pure quality. The plastic and glassware were cleaned by soaking in dilute HNO_3_ and were rinsed with deionized water prior to use. Standard metal solutions (1000 mg/L) were purchased from Merck (Darmstadt, Germany) or prepared in the laboratory from pure metals. The extractants were prepared as reported in Nemati et al. (2011).

### Texture and organic matter content in Rufiji sediments

About 6 g of freeze-dried sediment samples were first passed through a 63-μm sieve (US EPA 2010). The sand (˃ 63 μm size) content was determined gravimetrically. The fine fraction (< 63 μm) remaining was homogenized in 80 mL of 0.05% sodium hexametaphosphate (Centeri et al. 2015) with an ultrasonic stirrer for 3 min. Grain size distribution in the < 63 μm sediment suspension was determined with a Micromeritics SediGraph III for the amount (%) of silt and clay content. For estimating the organic matter in samples, about 0.2–0.5 g of freeze-dried samples were heated at 105 °C in an oven for 12 h. After cooling, the samples were stored inside a desiccator and weighed again, the samples were heated in a furnace at 550 °C for 4 h. The organic matter content was calculated as the total weight lost by the sample on heating (Heiri et al. 2001).

### ^210^Pb chronology

We applied the constant initial concentration (CIC model) to trace the down-core excess ^210^Pb (^210^Pb_ex_) profile. The CIC model assumes constant initial concentration of ^210^Pb_ex_ in surface sediments that is independent of the sedimentation rate (Krishnaswamy et al. 1971). With this model, the sedimentation rate can be calculated using the slope derived from linear regression of ln ^210^Pb_ex_ and the depth (x) according to Eq. 1 (Appleby and Oldfield 1983)

1 $$ \mathrm{C}={\mathrm{C}}_{\mathrm{o}}{\mathrm{e}}^{-\mathrm{kr}} $$

where C_o_ is the ^210^Pb_ex_ concentration (Bq kg^−1^d m) in surface sediment, k is the radioactive decay constant (0.03114 year^−1^), and r is the sedimentation rate. The age (t) of a sediment layer with ^210^Pb concentration C (Bq kg^−1^d.m) is determined based on Eq. 2

2 $$ \mathrm{t}=\frac{1}{\mathrm{k}}\ln \frac{\mathrm{C}\left(\mathrm{o}\right)}{\mathrm{C}} $$

**Fig. 2:**
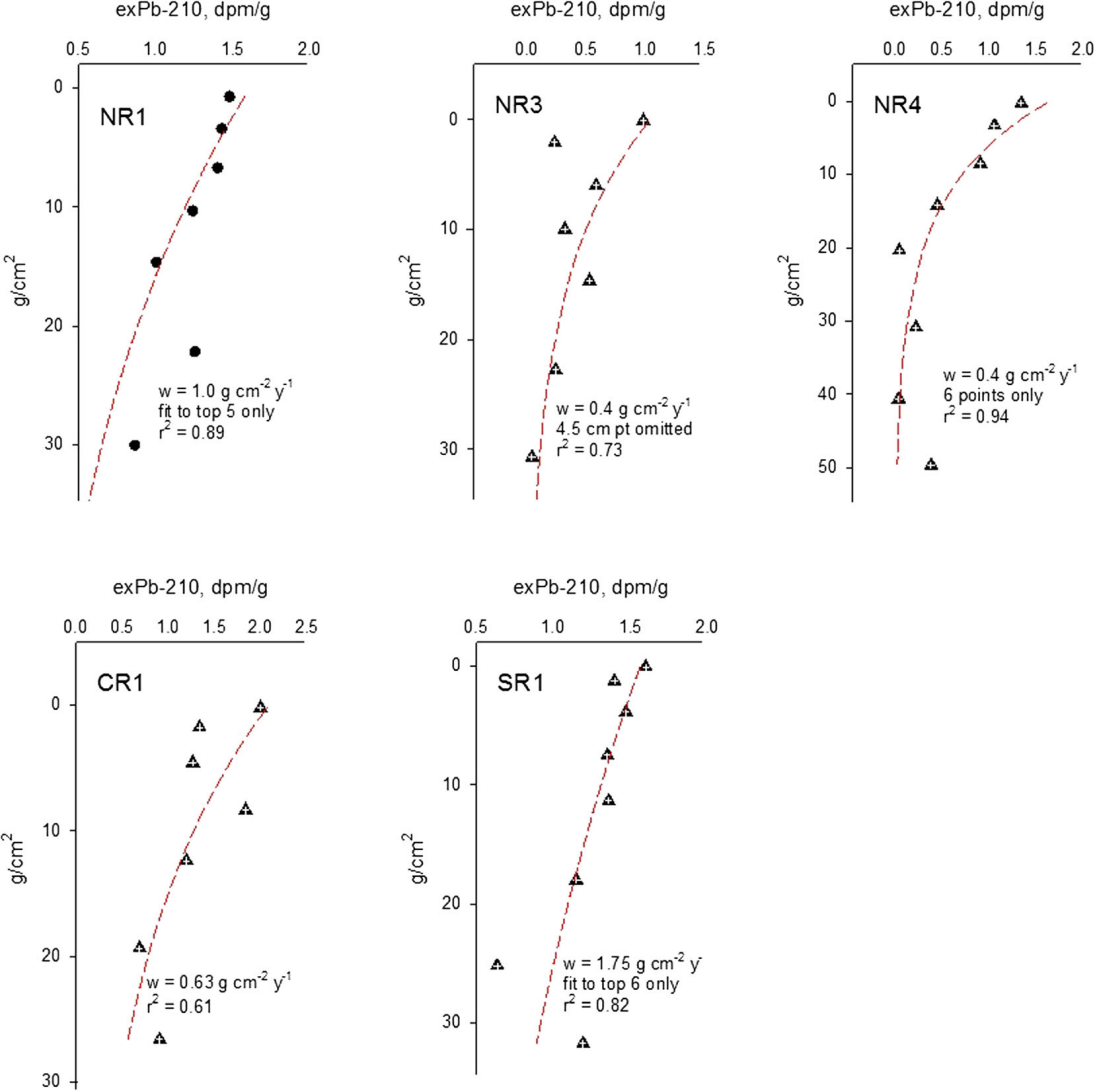
^210^Pb-based geochronology in the Rufiji delta sediment cores

The sediment age was estimated based on the slope of the line to calculate the sedimentation rate (cm year^−1^). To account for compaction in the layers, linear regression of the natural logarithm of ^210^Pb_ex_ (dpm/g) in dry sediments vs. the cumulative dry mass (g cm^–2^; Fig. 2) was calculated from bulk density of the sediment layer versus depth. This was used to estimate the sediment accumulation rate, (w) as gram per square centimeter per year.

The sediments were dated at the University of Wisconsin-Milwaukee by the ^210^Pb method. Prior to analysis, all samples were stored in sealed containers in the laboratory for more than 1 year. The ^210^Pb geochronology analytical procedure takes advantage of the fact that, within 2 years, secular equilibrium is achieved between ^210^Pb and its granddaughter ^210^Po in sediments, so that both isotopes have the same activity level. After drying and homogenization, the ^210^Pb activity was determined the chronology (Fuller et al. 1999; Krishnaswamy et al. 1971). In short, the procedure involved addition of ^209^Po as a tracer to the sediments to determine the chemical recovery and plating efficiency. The dried sediment (ca. 1 g) was then digested in 6 N HCl acid for 30 min at 95 °C, allowed to cool briefly, and four sequential 1 mL aliquots of 30% hydrogen peroxide and 1 drop of octanol were added at an interval of 30 min to oxidize the organic matter. Samples were then digested for 4 h in 6 N HCl and allowed to cool overnight. The samples were filtered to remove sediment and the solution reduced in volume by heating. Sample volume was then brought up to 50 mL with E-pure water; the pH was adjusted up to 1.0 by adding 100–200 mg of ascorbic acid and the solution was transferred to a plastic bottle. Polonium was plated onto one side of a copper disk at 95 °C in an oven. The plates were counted for 60,000 s on an Ortec dual-alpha spectrometer interfaced with a multi-channel analyzer. The samples were dated up to a depth of 30 cm in the core.

### Metal analyses

Metal analysis in mangrove sediments is based on the BCR sequential extraction method to determine trace metals concentrations in different geochemical fractions (e.g., carbonate, sulfidic, organic, oxide, or residual phases). Minor modification was made to the existing protocol and 7 M HNO_3_ was used instead of aqua-regia for extraction of trace metals in the residual fraction and for pseudo-total extraction (Borgese et al. 2013; SIS 2003). Certified reference sediments (CRM-601) and duplicate samples were used to test the accuracy and efficiency of the extraction procedure, and reproducibility of our results. Two blanks (procedural blank and step blank) were analyzed with each set of 10 samples to evaluate the results.

An internal check was performed on the results of the sequential extraction method by comparing the total amount of metals extracted by different reagents during the sequential extraction procedure with results of the pseudo-total digestion. The recovery of the sequential extraction method was calculated using Eq. 3 as reported in Nemati et al. (2011):

3 $$ \%\mathrm{Recovery}=\frac{\sum \left(\mathrm{F}1+\mathrm{F}2+\mathrm{F}3+\mathrm{F}4\right)}{{\mathrm{T}}_{\mathrm{p}}}\times 100 $$

where F1, F2, F3, and F4 were the different fractions and T_p_ was the pseudo-total metal concentration as per the BCR extraction scheme. Analytical results obtained for the reference materials differed by < 10% from certified values (Table 1).

**Table 1:** Concentration of trace metals in CRM-601 (Rauret et al. 1999) and those obtained in the present study

Trace metal	Step 1: TM concentration (mg/kg)				Step 2: TM concentration (mg/kg)				Step 3: TM concentration (mg/kg)			
	BCR	95% Confidence	Present Study	Within confidence	BCR	95% Confidence	Present Study	Within confidence	BCR	95% Confidence	Present Study	Within confidence
Cd	4.14	0.45	4.45	Yes	3.08	0.33	2.67	No	1.83	0.39	1.73	Yes
Cr	0.36	0.08	0.41	Yes	1.43	1.96	1.05	Yes	18.3	8.76	20.4	Yes
Cu	8.32	0.90	16.9	No	5.96	6.27	5.99	Yes	116	51.0	131	Yes
Pb	2.68	0.69	2.63	Yes	33.1	19.6	32.4	Yes	109	25.5	122	Yes
Ni	8.01	1.43	15.3	No	6.05	2.16	6.45	Yes	8.55	2.04	7.31	Yes
Zn	264	9.80	237	No	182	21.6	151	No	137	58.8	146	Yes

#### Exchangeable (water and acid soluble) fraction (F1)

About 0.5 g of accurately weighed sediment sample was extracted with 20 mL of 0.11 M acetic acid and shaking it for 16 h that was followed by centrifuging the sample at 3000 *g* for 20 min at 20 °C. After centrifugation, the supernatant was carefully decanted and stored for further analyses. The residue was washed with 60 mL of distilled water by shaking for 15 min, centrifuged, and the washings discarded.

#### Reducible (iron/manganese oxide) fraction (F2)

The residue from F1 fraction left was extracted with 20 mL of 0.11 M hydroxylamine hydrochloride (pH 2.0), and after shaking it for 16 h, the extract was filtered. The samples were centrifuged for 20 min at 3000×*g* and 20 °C. After centrifugation, the supernatant was carefully decanted and stored for analyses. The residue was washed with 60 mL of distilled water by shaking for 15 min, centrifuged, and the washings discarded.

#### Oxidizable (organic and sulfide) bound fraction (F3)

To the residue from F2 fraction, 5 mL of 8.8 M H_2_O_2_ was added in small aliquots and left for 1 h at room temperature to react. Another 5 mL of H_2_O_2_ was added and heated at 85 °C in a water bath. Afterwards 25 mL of 1 M ammonium acetate (pH 2.0) was added to the reaction mixture, and it was extracted after 16 h of shaking. The samples were centrifuged for 20 min at 3000 *g* and 20 °C. After centrifugation, the supernatant was carefully decanted and stored for further analyses. The residue was washed with 60 mL of distilled water by shaking for 15 min, centrifuged, and the washings discarded.

#### Residual bound fraction (F4)

To the residue from F3 fraction, 10 mL of 7 M HNO_3_ was added, and the reaction mixture was placed in an autoclave at 121 °C and 200 kPa for 30 min. After cooling, the samples were centrifuged for 20 min at 3000 *g* and 20 °C. The supernatant was carefully decanted and stored for further analyses and the remaining sediments were discarded.

#### Pseudo-total dissolution

Of sediment, 0.5 g was weighed and 10 mL of 7 M HNO_3_ was added and the reaction mixture was autoclaved at 121 °C and 200 kPa for 30 min (SIS 1993). After cooling, the samples were centrifuged for 20 min at 3000 *g* and 20 °C. After centrifugation, the supernatant was decanted carefully and stored in a 50-mL acid washed tube; the remaining sediment was discarded.

The mineral fractions obtained during each extraction step (fractions 1 through 4) and pseudo-total metal concentrations were analyzed on an ICP-MS (Perkin Elmer NexION 350). Prior to analysis, an internal standard was added, and the samples were diluted with deionized water. To evaluate the reliability of the extraction procedure, the reference standard CRM-601 was extracted, and analyzed for six certified trace metal concentrations in similar manner as the sediment samples.

### Trace metal enrichment in sediments

Anthropogenic input of trace metals in the Rufiji delta sediments was assessed by estimating the metal enrichment factor with respect to its background concentration. In absence of data on metal concentrations from pristine areas, the average continental crust (Rudnick and Gao 2003) or continental crustal abundances (Taylor and McLennan 1995) were used as background values of metals to calculate enrichment factors. Two approaches were used for comparing the metal enrichment factor at these sites in the Rufiji delta.

I_geo_ in this study was calculated using the geoaccumulation index proposed by Muller (1969). This index (Igeo) of trace metal is calculated by computing the base 2 logarithm of the measured total concentration of the metal over its background concentration (Abrahim and Parker 2008)

4 $$ {\mathrm{I}}_{\mathrm{geo}}={\log}_2\left(\frac{{\mathrm{C}}_{\mathrm{x}}}{1.5{\mathrm{B}}_{\mathrm{x}}}\right) $$

where C_x_ was the concentration of analyte “x” in sediment, B_x_ was the geochemical background concentration of analyte “x,” and 1.5 was the factor used to include possible variations that were due to lithological variations (Mohiuddin et al. 2010). Samples were considered unpolluted (I_geo_ ≤ 0), unpolluted to moderately polluted (0 ≤ I_geo_ ≤ 1), moderately polluted (1 ≤ I_geo_ ≤ 2), moderate to strongly polluted (2 ≤ I_geo_ ≤ 3), strongly polluted (3 ≤ I_geo_ ≤ 4), strongly to extremely polluted (4 ≤ I_geo_ ≤ 5), and extremely polluted (I_geo_ ≥ 5) according to Farkas et al. (2007).

Enrichment factor (EF) involved normalization of trace metal concentrations with respect to a reference metal such as Zr, Al, or Fe. Both Al and Fe have relatively high natural concentrations, and are therefore not expected to be substantially enriched by anthropogenic activities in sediments. EF was calculated in the sediments according to Aprile and Bouvy (2008):

5 $$ \mathrm{EF}={\frac{\raisebox{1ex}{${\mathrm{C}}_{\mathrm{xm}}$}\!\left/ \!\raisebox{-1ex}{${\mathrm{B}}_{\mathrm{xm}}$}\right.\left(\mathrm{sediment}\right)}{\raisebox{1ex}{${\mathrm{C}}_{\mathrm{xm}}$}\!\left/ \!\raisebox{-1ex}{${\mathrm{B}}_{\mathrm{xm}}$}\right.\left(\mathrm{background}\right)}}^{,} $$

where C_xm_/B_xm_ (sediment) was the metal concentration in relation to Al or Fe in sediment; C_xm_/B_xm_ (background) was the metal concentration in relation to Al or Fe in the crust (background). Based on EF, the degree of trace metal pollution was classified into seven classes namely: EF < 1 indicated no enrichment of metals; EF 1 to < 3 indicated minor enrichment; EF 3 to < 5 indicated moderate enrichment; EF 5 to < 10 indicated moderate to severe enrichment; EF 10 to < 25 indicated severe enrichment; EF 25 to < 50 indicated very severe enrichment, and EF 50 and above indicated extremely severe enrichment.

## Results

### Sediment chronology

Even though the ^210^Pb activity is relatively low in most of these cores, the data can be used to model age-depth relationship and estimate sedimentation rates. The ^210^Pb dates have been extrapolated to the upper 200 cm and the NR1, CR1, and SR1 sites are young dating back up to the 1970s at 30 cm. In contrast, sediments at NR3 and NR4 sites extend to the 1880s at same depth. The measured ^210^Pb activity in the Rufiji delta mangrove sediments does not reach the background level at NR2, and hence the age could not be validated at this site. Sedimentation rate ranges from 0.4 at NR3 and NR4 sites to 1.75 g cm^−2^ year^−1^ at SR1 site, respectively (Fig. 2). The sedimentation rates are 0.14 cm year^−1^ at NR1, 0.05 cm year^−1^ at NR3, 0.04 cm year^−1^ at NR4, 0.10 cm year^−1^ at CR1, and 0.28 cm year^−1^ at SR1 sites (Fig. 2b). Sediment age in the deeper cores was extended to a maximum value of ca. 540 year by extrapolation.

### Grain size

**Fig. 3:**
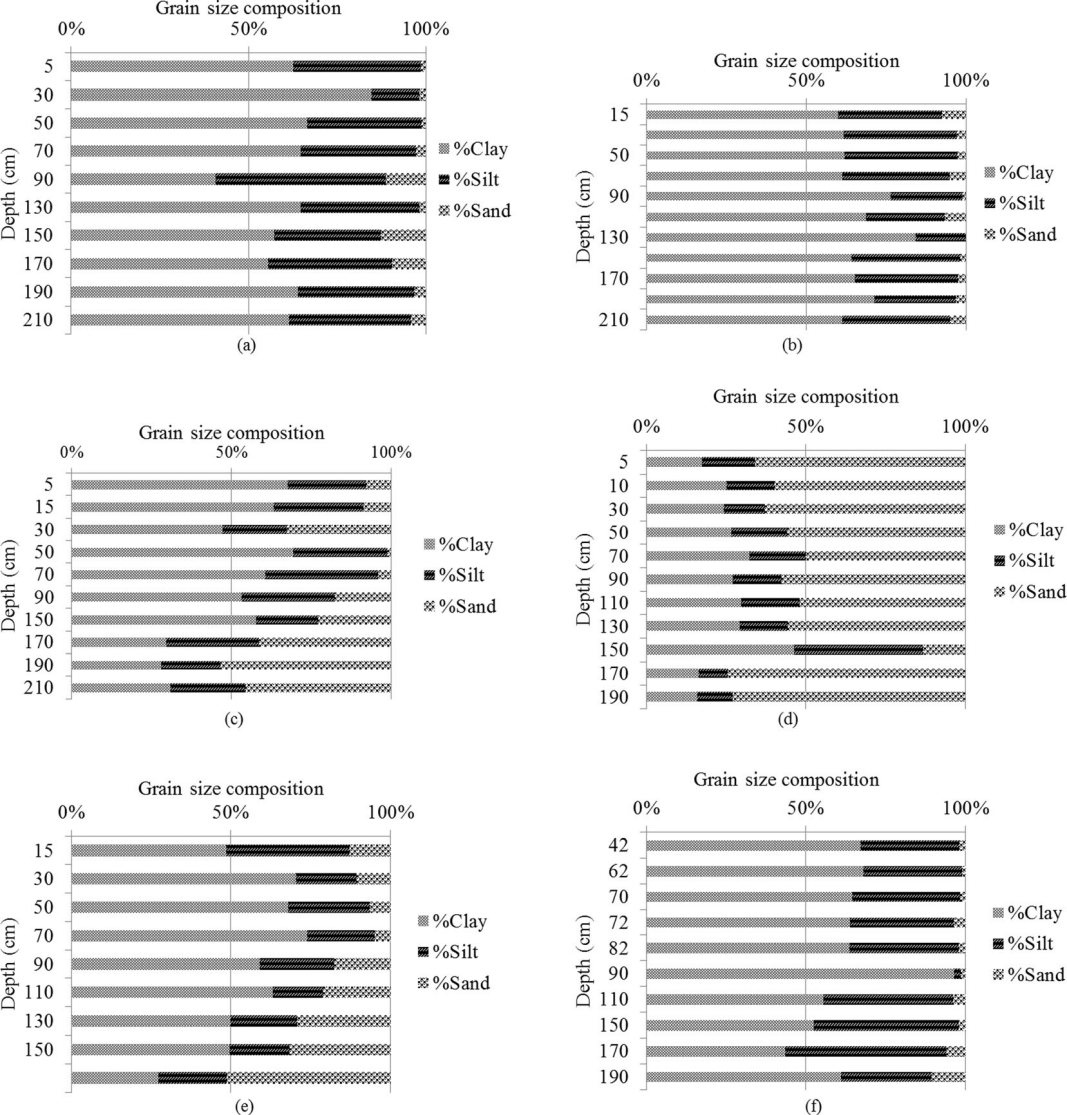
The distribution of clay, silt, and sand at (**a)** NR1, **(b)** NR2, **(c)** NR3, **(d)** NR4, **(e)** CR1, and **(f)** SR1 sites in the Rufiji delta mangrove sediments

Figure 3 summarizes the results for grain size distribution in sediment cores from the Rufiji mangroves. The grain size distribution at sites NR1, NR2, and SR1 shows similar pattern. These sites have high clay content (~ 50%), followed by silt and sand. The percentage of fine grains (clay + silt) in the sediments is in the order NR1 ≥ SR1 > NR2. At NR3, clay particles dominate followed by silt and sand; the highest sand content (about 40 to 50%) occurs at depth of 150–210 cm. At this site, the sand content increases considerably compared to the other three sites. Similarly, site NR4 has higher sand than clay and silt content. Sediments at site CR1 have high clay (45%) followed by silt and sand content. The overall order of decreasing fine-grain sediment content in the cores is NR1 ≥ SR1 > NR2 > CR1 > NR3 > NR4.

### Organic matter content

**Fig. 4:**
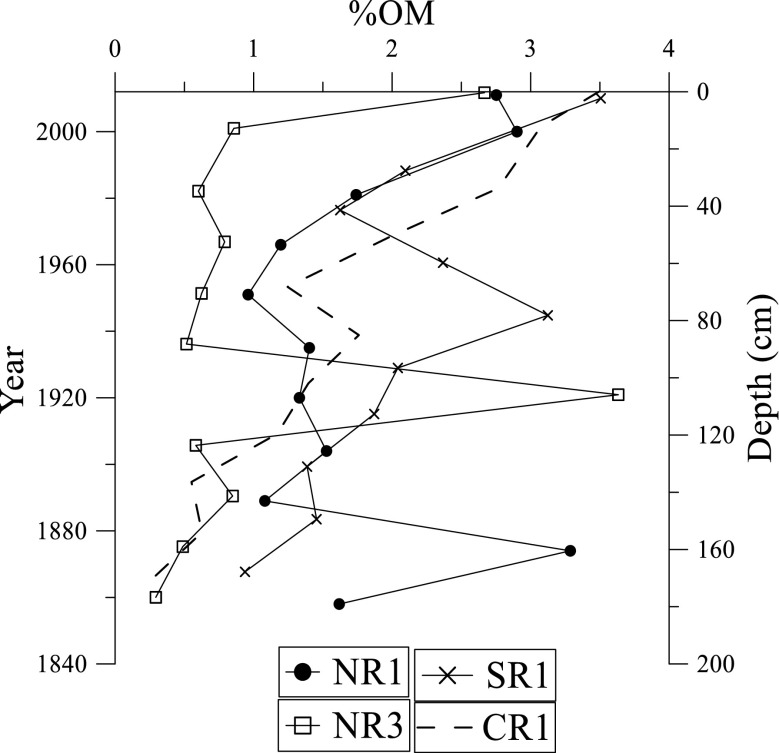
Variation of organic matter content in the Rufiji delta mangrove sediments

Organic matter (OM) content at the NR1 site ranges from 0.9% at 80 cm to 4.8% at 40 cm depth (Fig. 4). The NR2 site has similar trend for OM distribution as in NR1. OM at NR2 site ranges from 1.1% at 15 cm to 2.3% at 40 cm. No definite trend is observed for these two sites. Instead, several outliers are observed. For NR3, NR4, CR1, and SR1 sites, OM content increases core upwards. NR3 site has OM ranging from 0.6% at 200 cm to 7.3% at 40 cm. NR4 has OM content, ranging from 0.2% at 170 cm to 2.1% at 1.0 cm. The OM content at CR1 site ranges from 0.4% at 200 cm to 5.7% at 5 cm. In SR1 site, OM content ranges from 0.7 at 200 cm depth to 3.8% at 5 cm depth.

### Distribution of trace metals in sediments

**Fig. 5:**
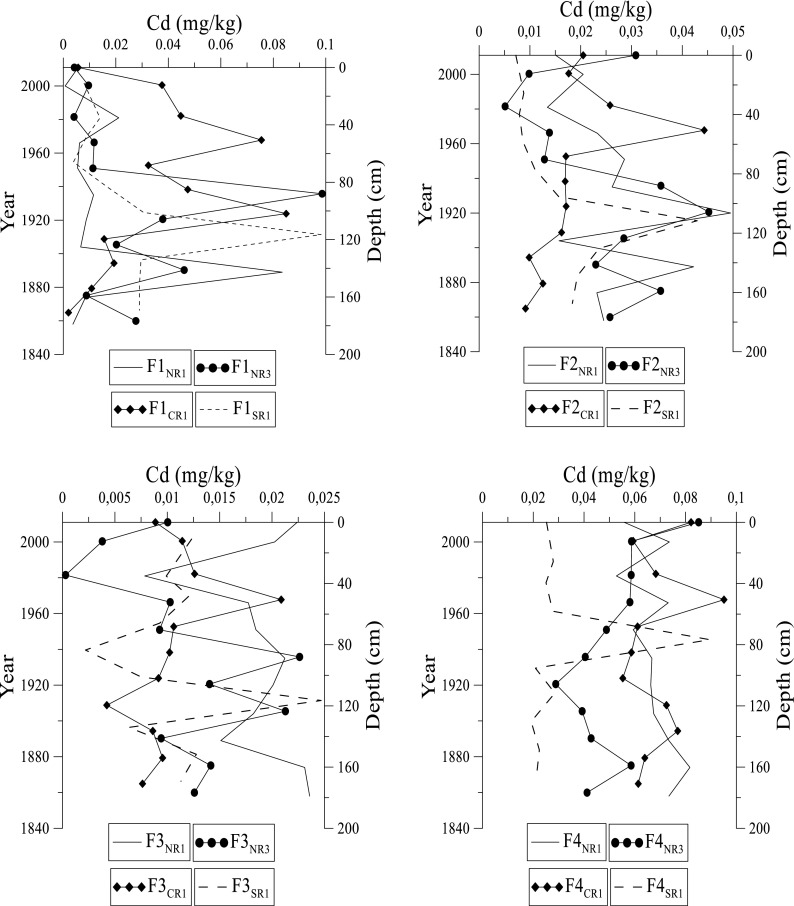
Distribution of cadmium at NR1, NR3, CR1, and SR1 sites in fractions 1 to 4 in the Rufiji delta sediments

**Fig. 6:**
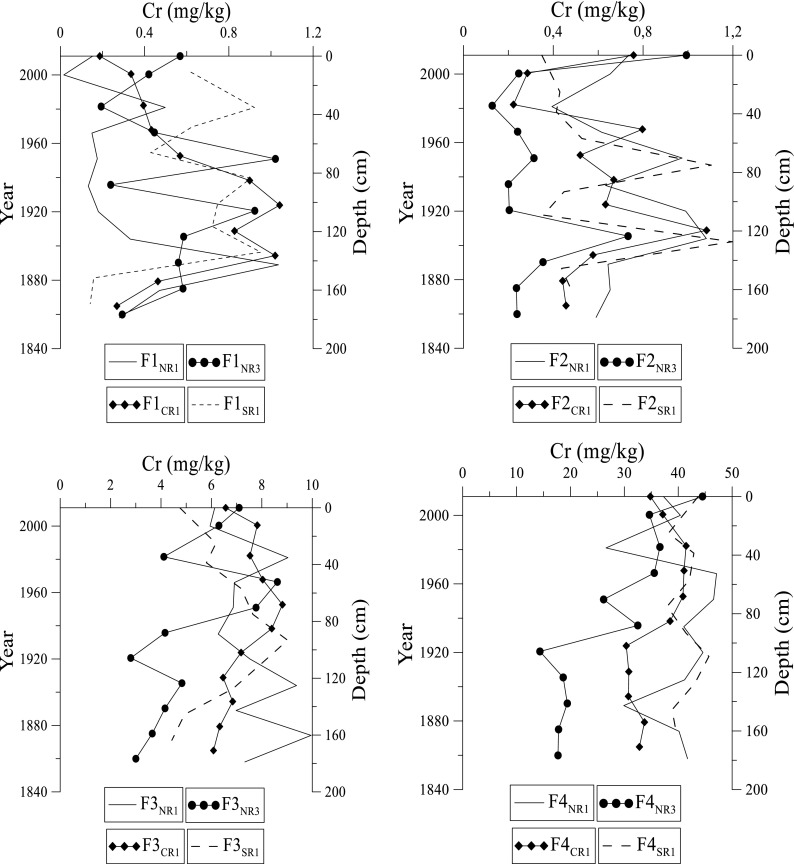
Distribution of chromium at NR1, NR3, CR1, and SR1 sites in fractions 1 to 4 in the Rufiji delta mangrove sediments

**Fig. 7:**
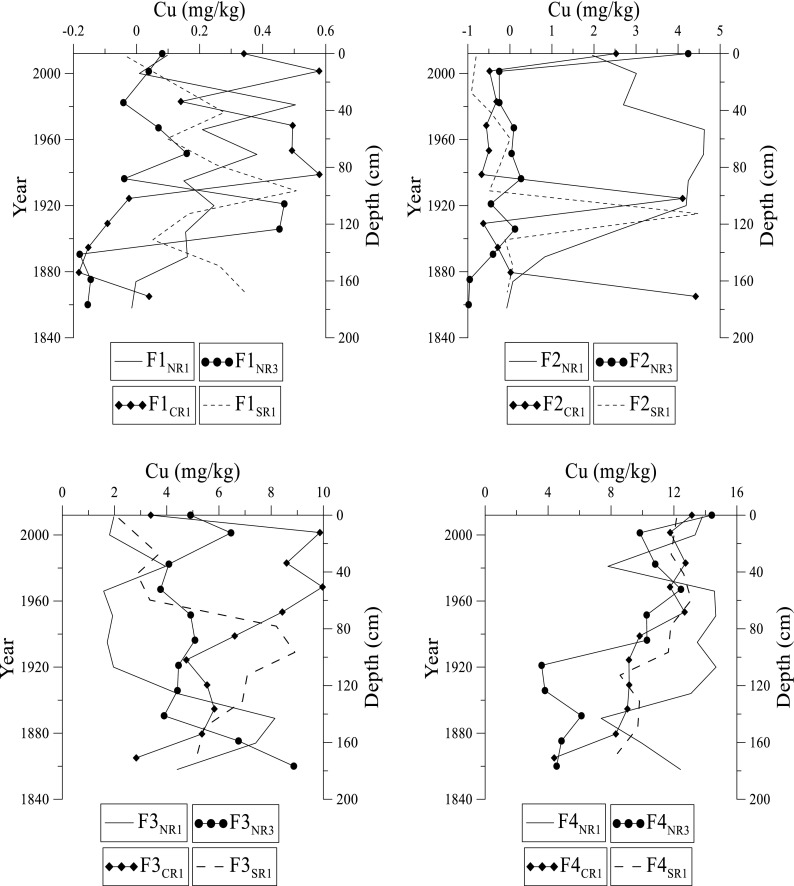
Distribution of copper at NR1, NR3, CR1, and SR1 sites in fractions 1 to 4 in the Rufiji delta mangrove sediments

**Fig. 8:**
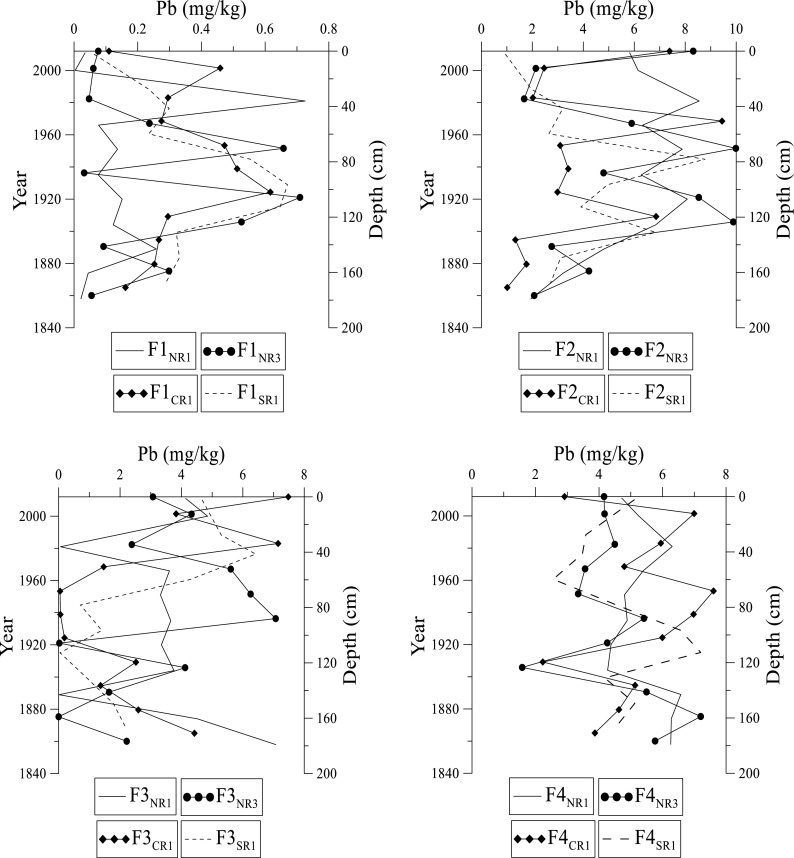
Distribution of lead at NR1, NR3, CR1, and SR1 sites in Fractions 1 to 4 in the Rufiji delta mangrove sediments

**Fig. 9:**
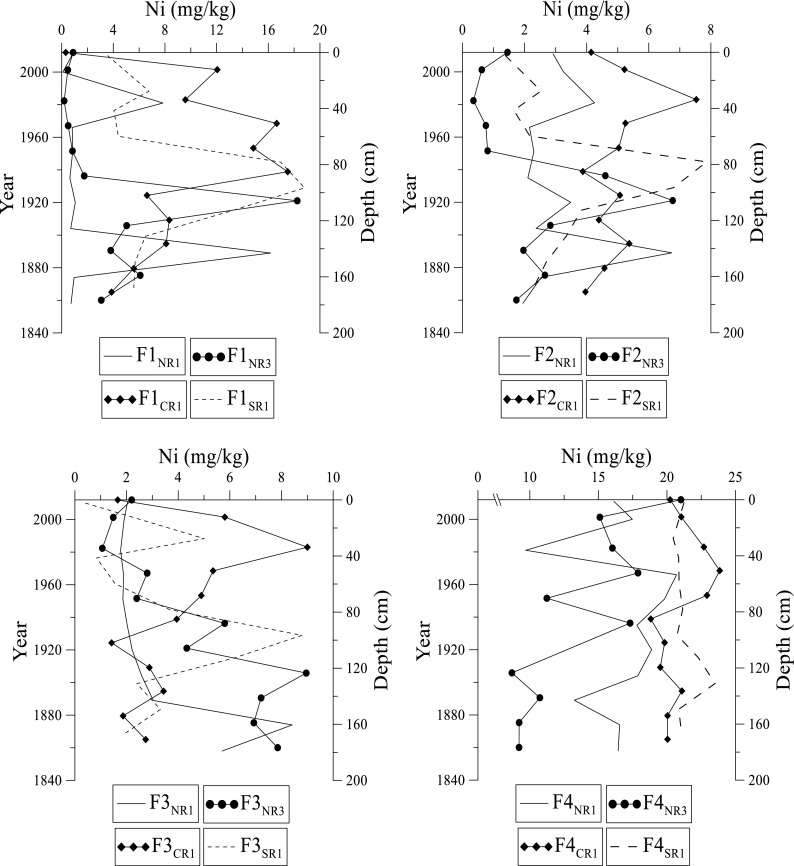
Distribution of nickel at NR1, NR3, CR1, and SR1 sites in fractions 1 to 4 in the Rufiji delta mangrove sediments

**Fig. 10:**
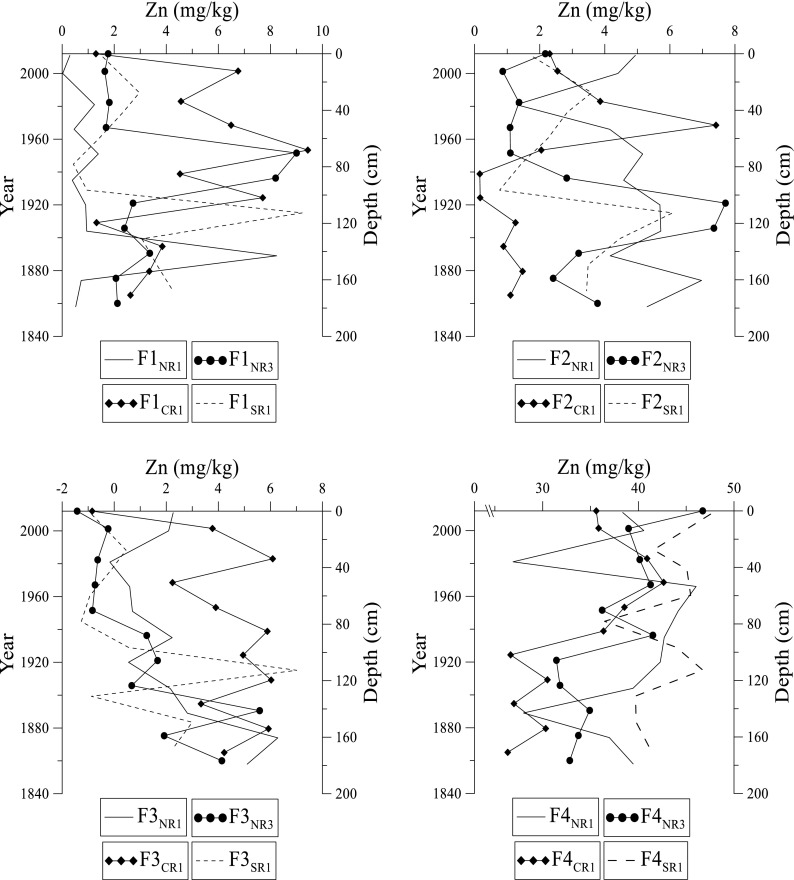
Distribution of zinc at NR1, NR3, CR1, and SR1 sites in fractions 1 to 4 in the Rufiji delta mangrove sediments

Trace metal concentrations in this study were compared to the certified trace metal values for the standard CRM-601. The results observed are generally within range (except Cu and Zn) confirming the extraction efficiency and reliability of our data (Table 1). The % recovery of trace metals in the extractions is > 95%. The distribution of specific trace metals (Cd, Cr, Cu, Pb, Ni, and Zn) in various fractions in the Rufiji sediments is presented in Figs. 5, 6, 7, 8, 9, and 10. Sites NR1 and NR2 have similar physical and chemical characteristics (except the dominant vegetation cover). Therefore, results from the NR1 site are presented in this study as a representative profile. Likewise, NR3 and NR4 sites have similar trends, and hence results from site NR3 are presented as a representative profile.

Cadmium (Cd) in the F1 fraction increases in concentration from surface to 100 cm depth at NR3, CR1, and SR1 sites (Fig. 5). The NR1 site has the highest Cd concentration at 160 cm. Cd concentrations at NR1, NR3, CR1, and SR1 sites range up to 0.10 mg kg^–1^ in the F1 fraction. The highest concentration of Cd occurs between 40 and 120 cm at NR1, NR3, CR1, and SR1 sites. These depth intervals correspond to the period 1910–1980. In F2 fraction, the concentration of Cd ranges from 0.01 to 0.05 mg kg^–1^. There is minimal change in distribution of Cd with depth except at 40 cm (CR1 site) and 90 cm (NR1, NR3, and SR1 sites) depths that correspond to 1970 and 1920 when Cd concentrations increase sharply. In F3 fraction, Cd is ≤ 0.02 mg kg^–1^, and concentration decreases slightly with age. The highest concentration of Cd occurs at 50 cm in CR1, 90 cm at NR3, 110 cm at SR1, and 180 cm at NR1 site. These depths correspond to the years 1970, 1930, 1910, and 1860, respectively, in these cores. Cadmium in F4 fraction at these sites ranges from 0.05 to 0.09 mg/kg and indicates a gradual fluctuation in concentration. The overall trend for Cd in fractions 1 through 4 indicates a decreasing order F1 > F4 > F2 > F3.

Chromium ranges from 0.09 to 0.22 mg kg^–1^ (Fig. 6). The concentration of Cr in F1 fraction is high towards the lower part or mid-section of the core (Fig. 6). Elevated concentration of Cr occurs at SR1, whereas at NR1, concentration decreases. At NR1 and CR1 sites, Cr increases in concentration towards the lower half of the core and this is followed by a steady decrease with depth. Cr at NR3 has a uniform distribution until it reaches 50 cm, after which Cr increases in concentration. In F2 fraction, Cr shows high concentration in surface sediments followed by alternating decrease and/ increase of Cr at different depth intervals. Similarly, in the F3 and F4 fractions, Cr has the same trend and decreases with depth. The overall trend for Cr in fractions 1 through 4 indicates a decreasing order F4 > F3 > F2 > F1.

Copper ranges from 0.01 to 15.1 mg kg^–1^ in the Rufiji sediments (Fig. 7). The concentration is low at NR3 compared to the other sites. The variation of Cu in F1 fraction increases from the top to about 100 cm at all sites followed by a gradual decrease in its concentration. In F2 fraction, between the different sites, NR1 has the highest concentration that increases around 1960 at all sites. In F3 fraction, Cu gradually increases in concentration just around the mid-section before it decreases. In F4 fraction, Cu is more enriched near the top and surface sediments; Cu decreases with depth at different sites except at NR1.

Lead in the Rufiji sediments ranges from 11.5 to 17.0 mg kg^–1^ (Fig. 8). The concentration of Pb in F1 fraction slightly increases with depth, and the highest concentration at these sites occur around 40, 80, and 100 cm, respectively (Fig. 8). There is a gradual decrease in Pb with depth at all sites, but a few outliers exist. In the F2 fraction, at NR1 and CR1 sites, the highest concentration of Pb occurs at 40 cm. The NR3 and SR1 sites have the highest concentration at 60 and 90 cm, respectively. In F3 fraction, Pb is high in the upper half of the core, but gradually decreases with depth. In F4 fraction, Pb shows less variation with depth unlike the other fractions. The highest concentration for Pb in F4 fraction occurs at 160 cm for both NR1 and NR3 sites, 60 cm at CR1 site, and at 100 cm in SR1 site. The overall trend for Pb in fractions 1 through 4 indicates a decreasing order F2 > F4 > F3 > F1. The average concentration of Pb at NR1, NR3, and CR1 sites is found to be statistically same, but different from SR1.

Nickel in the Rufiji delta sediments ranges from 0.20 to 23.8 mg kg^–1^ (Fig. 9). In F1 fraction, Ni significantly increases with depth from the surface to 100 cm followed by its decrease at NR3, CR1, and SR1 sites. Ni has high concentration up to 160 cm at NR1. In F2 fraction, the highest concentrations are at 160 cm in NR1. The NR3, CR1, and SR1 sites indicate a gradual increase in concentration in surface sediments down-core. The highest concentration in this fraction occurs at 20 cm (CR1), 60 cm (SR1), and 100 cm (NR3). Concentration of Ni is relatively high in the F4 fraction compared to F1, F2, and F3 fractions (Fig. 9).

Zn ranges from 0.01 to 47.5 mg kg^–1^ in these sediments (Fig. 10). Abundance of Zn at these sites is in the order NR1 > SR1 > CR1 > NR3 (Fig. 10). Distribution of Zn in F1 fraction increases a little from sediments near the top to 60 cm depth at CR1 and to 100 cm at NR3 and SR1 sites. There is no significant variation at NR1 site up to a depth of 140 cm and then Zn increases in concentration. Below 80–120 cm, Zn shows a gradual decrease at all sites. In F2 fraction, the concentration of Zn at NR3 and CR1 sites increases with depth to its highest level at about 100 and 40 cm, respectively. At NR1 and SR1 sites, the concentration of Zn slightly increases with depth. The F3 fraction has slightly higher Zn concentration with depth. The variation of Zn in F4 fraction indicates an overall decrease with depth. Zn is more enriched in the F4 fraction at NR1, NR3, CR1, and SR1 sites than in F1, F2, and F3 fractions.

### Enrichment assessment of trace metals

Enrichment assessment in the Rufiji sediments was calculated with respect to several elements (besides the six elements discussed in the BCR protocol) to provide a more comprehensive picture of metal contamination at these sites. The EF and geoaccumulation index calculated for assessment of trace metal pollution at the different sites are presented in Tables 2 and 3, respectively.

**Table 2:** Enrichment factor of trace metals in the Rufiji mangrove sediments

	As	Cd	Cr	Co	Cu	Fe	Pb	Mn	Ni	V	Zn
NR1	8.34	2.88	0.96	2.03	1.87	2.25	2.55	1.87	1.16	1.40	1.82
NR2	7.22	0.75	1.07	1.54	2.21	2.2	2.98	2.97	1.09	1.52	1.77
NR3	13.4	3.52	1.22	1.49	2.28	2.66	2.99	1.60	1.38	1.87	2.28
NR4	7.22	3.85	1.32	1.81	2.50	1.89	3.12	2.58	1.15	1.79	2.44
CR1	10.4	2.91	0.95	1.49	1.90	1.70	2.21	0.82	1.04	1.42	1.66
SR1	7.89	4.22	1.19	2.13	2.78	2.06	2.72	1.26	1.22	1.95	2.18

**Table 3:** Geoaccumulation index (I_geo_) of trace metals in the Rufiji mangrove sediments

Site	As	Cd	Cr	Co	Cu	Fe	Pb	Mn	Ni	V	Zn
NR1	0.34	− 0.12	− 0.59	− 0.27	− 0.3	− 0.22	− 0.17	− 0.3	− 0.51	− 0.43	− 0.32
NR2	0.21	− 0.77	− 0.62	− 0.46	− 0.3	− 0.31	− 0.17	− 0.17	− 0.61	− 0.46	− 0.4
NR3	0.30	− 0.28	− 0.74	− 0.65	− 0.47	− 0.4	− 0.35	− 0.62	− 0.69	− 0.55	− 0.47
NR4	− 0.06	− 0.33	− 0.8	− 0.66	− 0.52	− 0.64	− 0.43	− 0.51	− 0.86	− 0.67	− 0.53
CR1	0.36	− 0.19	− 0.68	− 0.48	− 0.37	− 0.42	− 0.31	− 0.74	− 0.64	− 0.5	− 0.43
SR1	0.23	− 0.04	− 0.59	− 0.33	− 0.22	− 0.35	− 0.23	− 0.56	− 0.58	− 0.37	− 0.33

## Discussion

### Deposition and physical characteristics

The Rufiji mangrove sediments show variability in sedimentation rate and chronology. Sites cores from NR1 and SR1 sites have the lowest sedimentation rate amongst the six sampled stations because these sites are inundated by tides, and they receive considerable amount of runoff water resulting in erosion and loss of sediments. Other factors contributing to the low sedimentation rates at these sites could be due to (1) physical and biological mixing at or near the sediment-water interface in surface sediments resulting in a flat ^210^Pb activity profile versus depth (Tylmann 2004), and (2) chemical diffusion or advection within the pore water at these sites (Gonçalves et al. 2012).

At NR3, clay particles dominate followed by silt and sand. Core NR3 has the highest sand content (40 to 50%) at 150–210 cm depth. At this site, the sand content increases considerably compared to the other three sites. Similarly, site NR4 has higher (ca. 55%) sand than clay and silt content. Sediments at CR1 site have higher (ca. 45%) clay content than silt and sand. The overall order of decreasing fine-grain sediment content in the core is NR1 ≥ SR1 > NR2 > CR1 > NR3 > NR4. A possible reason for such a trend is that NR1 and SR1 sites receive considerable amount of land-based run-off, which carries fine-grain sediments further downstream. In addition, location of these sampling sites with respect to the higher mangrove vegetation cover (density) has an influence on the grain size distribution pattern because it reduces the effect of erosion due to precipitation (Ewane and Heon 2016).

### Chemical characteristics in mangrove sediments

Average OM content in Rufiji sediments has the highest (26.2%) value at CR1 and the lowest (5.8%) at NR4 site. In general, OM distribution in these sediments is closely associated with sediment texture and clay content. The presence of high OM content at CR1 and SR1 sites indicates input of OM from upstream run-off waters. These sites are affected by flooding during high tides, and water from the Indian Ocean enriched in marine OM is deposited at sites CR1 and SR1. The NR3 site is currently a semi-desert and vegetation dieback and accumulation of plant matter in sediments results in high OM content at this site. Sites NR1, NR3, and NR2 are also influenced by anthropogenic activities such as logging, burning of charcoal, and farming, which contribute to the OM content at these sites. The sediment grain size rich in clay and silt fractions reflects that the large surface area of fine particles (sediment-specific surface area) results in greater sorption of OM to sediments. In addition, variation of OM content with depth is also affected by mineralization as part of the early diagenetic changes (Sanders et al. 2012), after the sediments are deposited.

The distribution of trace metals in different sediment fractions is based on the well-established BCR protocol. The protocol involves sequential chemical extraction to progressively leach out metals sorbed to the sediment matrix and assess their mobility. A one-way ANOVA tests shows that trace metal concentrations are significantly different between the stations (*p* < 0.005). The spatial variability could be due to the variation of mineralogical composition in sediments at the sampling sites or metals that are released from different sources. Fluvial influence on sediment transport plays an important role in trace metal distribution. As tidal currents increase, sediment particles are lifted into the water column and transported downstream towards the delta, particularly to those sites that are more exposed to sediment discharge from the Rufiji River. The distribution of trace metals at these sites is also influenced by OM content and Fe-Mn co-precipitation, and formation of insoluble precipitates that differ between the sites.

The relative contribution of Cd in F1 fraction in sediments is high compared to its distribution in other fractions. The dominance of lattice-bound Cd fraction (F4) over F2 and F3 fractions may be due to the facts that the Rufiji River erodes and carries Cd-rich rock fragments and sediments from upstream that are later trapped in the mangroves. The overall Cd concentration is in the order F1 > F4 > F2 > F3. The sediment characteristics and fractionation in the Rufiji sediments suggests that Cd could be available for biological uptake at this site because it is mainly associated with the acid leachable (F1) fraction. Cadmium is slightly enriched in top sediments, which may be influenced by the particle size of sediments (Zhao et al. 2010) and OM content. The spatial distribution trend shows that SR1 > CR1 > NR3 > NR1 Cd concentrations. While the highest Cd concentration occurs in SR1, significant difference between the sites is absent.

Chromium is most enriched at the NR1 site. The presence of Cr in F1 and F2 fractions is most probably related to anthropogenic activities in upstream sections of the Rufiji River. In addition, Cr also occurs in the F3 fraction. Consistent with this, good correlation between the F3 fraction and organic carbon in surface sediments suggests scavenging of Cr by OM. However, F4 fraction constitutes > 95% of total Cr suggesting that Cr is primarily lattice-bound, and it is available for limited biological uptake. The low concentration of Cr in certain samples could be due to the high sand content at these depths. The overall trend for Cr in F1 through F4 fractions shows the order NR1 > CR1 > SR1 > NR3. The NR1 site receives considerable amount of run-off water from upstream sites carrying with it traces of metals that originate from chemical weathering, atmospheric deposition, and anthropogenic activities that increase the Cr levels in sediments.

Copper has the same trend as Cd in the Rufiji delta. However, in this study, Cu is more enriched in the F4 fraction than F3 fraction, particularly in surface sediments even though Cu has higher affinity for OM, and readily forms complexes with humic substances (da Silva et al. 2014; Marchand et al. 2012). In general, Cu occurs in low concentration in the F1 and F2 fractions. Marchand et al. (2011) concluded that high metal concentration in the F3 fraction in mangrove sediments is due to the high OM content and anoxic conditions, which favor accumulation of metals in mangrove sediments. The overall spatial distribution for Cu in terms of abundance at different sites is in the order SR1 > NR1 > CR1 > NR3 sites.

Lead in these sediments reveals a moderately high concentration that is like other unpolluted places in East Africa (Christopher et al. 2014; Mwashote 2003; Ogoyi et al. 2011). Lead decreases with depth in the F1 through F4 fraction, and the highest concentration at all sites occur between 40 and 120 cm. Accumulation of Pb in surficial sediments is probably due to its relatively poor mobility and strong tendency to form metallo-organic complexes (Olade 1987). Consistent with this, spatial and temporal distribution of Pb is strongly associated with fine-grain sediments rich in OM content. Lead shows little association/affinity with Fe-Mn oxides in sediments. Distribution of Pb between the different sites decreases in the order SR1 > NR1 > CR1 > NR3 suggesting that the SR1 site is more affected by anthropogenic activities.

Nickel is mostly (≥ 90%) associated with the F4 fraction. It is less than the typical concentration of Ni in sediments. For example, Chukwuemeka et al. (2014) and Stephen and Oladele (2012) reported that Ni in coastal sediments ranges from 1.00 to 80.0 mg/kg. However, Cempel and Nikel (2006) reported that Ni content in sediments is variable and ranges from 3.00 to 1000 mg kg^–1^. The fact that ≥ 90% of Ni is associated with the F4 fraction, it suggests that Ni is perhaps not available for uptake by plants in the Rufiji sediments. On comparing the different sampling sites, it seems that the average concentration of Ni at CR1 shows significant differences with other sites (*p* < 0.05). This variability is mainly related to the OM content and grain size in sediments.

Zinc is enriched in surface sediments particularly in the F4 fraction. This suggests that deposition of Zn in sediments due to anthropogenic activities in the delta is minimal. CR1 has the highest Zn concentration, whereas the lowest concentration occurs in SR1. The spatial variation in Zn is probably due to dissolution of Zn derived from ocean that is carried by tides. Other than this, there is no significant statistical difference between the sites.

### Enrichment assessment of trace metals in the Rufiji delta mangrove sediments

The EF for As shows moderate to high enrichment in Rufiji sediments at the NR1, NR2, NR4, and SR1 sites (Table 2). The EF for Cd in sediments ranges from 0.75 to 4.2. At NR2, the EF value is below unity suggesting background concentration for Cd in these sediments. Cadmium is slightly enriched at the NR1 and CR1 sites (i.e., 2.88 and 2.91, respectively). At NR3, NR4, and SR1 sites, the EF values suggest moderate enrichment of Cd. The EF values for Cr ranges from 0.95 to 1.32 suggesting lack of enrichment in NR1 and CR1 and minor enrichment at sites NR2, NR3, NR4, and SR1. In case of elements Co, Cu, Fe, Ni, V, and Zn, the EF ranges from 1 to < 3. This suggests low enrichment of these metals in the Rufiji sediments. Lead is moderately enriched at NR4, and slightly enriched at the sites NR1, NR2, NR3, CR1, and SR1. Similarly, enrichemnt of Mn in CR1 core is low, but it is more abundant at the other sites.

The I_geo_ value for As ranges from 0 to 1 suggesting that mangrove sediments are uncontaminated to moderately contaminated (except sediments from NR4 which has I_geo_ value < 0). However, the results indicate that sediments are not contaminated in terms of Cd, Cr, Co, Cu, Fe, Pb, Mn, Ni, V, and Zn since the I_geo_ values are negative (Table 3).

## Conclusions

Trace metals in Rufiji sediments mainly occur in the residual phase (F4 fraction) at all sites. This suggests that these trace metals are of natural origin most likely arising from weathering of parent rocks in the catchment. The trends imply there is limited anthropogenic input that is further supported by the lack of high/enriched concentrations in recently deposited sediments from upstream sections. The metal concentrations are high at sites which have high OM content and fine-grain size. The EF and I_geo_ values for As are elevated in sediments from the Rufiji mangroves. The source for As enrichment at these sites is unclear. We recommend further studies in the Rufiji sediments with respect to pH and salinity differences that might affect their distribution locally.
